# Effect of Probiotic Consortium on the Local Inflammatory Process in Chronic Periodontitis

**DOI:** 10.5195/cajgh.2013.109

**Published:** 2014-01-24

**Authors:** Zhanagul Khasenbekova, Saule Saduakhasova, Alexandr Gulayev, Almagul Kushugulova, Samat Kozhakhmetov, Gulnara Shakhabayeva, Indira Tynybayeva, Talgat Nurgozhin, Zhaxybay Zhumadilov

**Affiliations:** Center for Life Sciences, Nazarbayev University, Astana, Kazakhstan

**Keywords:** probiotics, chronic periodontitis, lactobacilli

## Abstract

**Introduction:**

Inflammatory periodontal disease is one of the major concerns of researchers and clinicians, because it can lead to tooth loss and an increased risk of systemic pathologies, even at the age of 35. The purpose of this study was to determine the effects of gelatin-based probiotic consortium on the local and general factors of inflammation in rats with chronic periodontitis.

**Methods:**

The study object was a complex of probiotic bacteria based in an odourless 6% gelatin plate with neutral flavour. A cellular biomass of the consortium consists of following lactobacilli: *Lactobacillus casei subsp. pseudoplantarum, Lactobacillus caseisubsp.casei, L.fermentum,* and *L. helveticus*. The viable cell number was 2.5 × 109 CFU/ml. The model of chronic periodontitis was reproduced in the white random-bred rats that weighed 160–220g, by keeping them on a low-protein diet. After three months, symptoms associated with medium and severe chronic periodontitis were observed in the rats. Application was carried out on the oral mucosa of rats 1 time per day for 14 days. The stickers lacking consortium of microorganisms were used as the placebo. The “Solcoseril” gel was chosen as a comparator. The hematologic, biochemical, and morphological characteristics were investigated.

**Results:**

A complete clearance of periodontal pockets was observed during an objective examination of the experimental group rats on the 14^th^ day of the experiment. Moreover, a gingival mucous turned pink, and there were no cyanosis tissues. The local changes were accompanied by improvement in hematological parameters, such as a reduction of blood eosinophilia and neutrophilia, and a recovery of the white blood cells number to the normal degree within the group that received the probiotic complex. A decrease of the acute plethora of microvasculature was observed morphologically as a result of the treatment. There were signs of basal layer activation of the stratified squamous epithelium with a merger of the acanthosis outgrowths and a formation of the fibrotic nodules. Biochemical investigations did not show significant changes in the indicators.

**Conclusions:**

In the settings of the chronic periodontitis model, the use of gelatin-based probiotic consortium consisting of *Lactobacillus casei subsp. pseudoplantarum, Lactobacillus caseisubsp.casei, L.fermentum, L. helveticus*. at 2.5 × 109 CFU/ml viable cell numbers lead to the reduction of the local inflammatory manifestations of the periodontitis in 14 days of treatment.

